# Discovery of Anti-Amoebic Inhibitors from Screening the MMV Pandemic Response Box on *Balamuthia mandrillaris, Naegleria fowleri*, and *Acanthamoeba castellanii*

**DOI:** 10.3390/pathogens9060476

**Published:** 2020-06-16

**Authors:** Christopher A. Rice, Emma V. Troth, A. Cassiopeia Russell, Dennis E. Kyle

**Affiliations:** 1 Department of Cellular Biology, University of Georgia, Athens, GA 30602, USA; 2 Center for Tropical and Emerging Global Diseases, Athens, GA 30602, USA; Emma.Troth@uga.edu (E.V.T.); Antoinette.Russell@uga.edu (A.C.R.); 3 Department of Infectious Diseases, University of Georgia, Athens, GA 30602, USA

**Keywords:** MMV, Pandemic Response Box, phenotypic screening, drug discovery, *Balamuthia mandrillaris*, *Naegleria fowleri*, *Acanthamoeba castellanii*, antiparasitic agents

## Abstract

Pathogenic free-living amoebae, *Balamuthia mandrillaris*, *Naegleria fowleri*, and several *Acanthamoeba* species are the etiological agents of severe brain diseases, with case mortality rates > 90%. A number of constraints including misdiagnosis and partially effective treatments lead to these high fatality rates. The unmet medical need is for rapidly acting, highly potent new drugs to reduce these alarming mortality rates. Herein, we report the discovery of new drugs as potential anti-amoebic agents. We used the CellTiter-Glo 2.0 high-throughput screening methods to screen the Medicines for Malaria Ventures (MMV) Pandemic Response Box in a search for new active chemical scaffolds. Initially, we screened the library as a single-point assay at 10 and 1 µM. From these data, we reconfirmed hits by conducting quantitative dose–response assays and identified 12 hits against *B. mandrillaris*, 29 against *N. fowleri*, and 14 against *A. castellanii* ranging from nanomolar to low micromolar potency. We further describe 11 novel molecules with activity against *B. mandrillaris*, 22 against *N. fowleri*, and 9 against *A. castellanii*. These structures serve as a starting point for medicinal chemistry studies and demonstrate the utility of phenotypic screening for drug discovery to treat diseases caused by free-living amoebae.

## 1. Introduction

Pathogenic free-living amoebae (FLA) are highly lethal organisms whose under-recognized infections pose a significant risk to human health. *Balamuthia mandrillaris*, *Naegleria fowleri*, and *Acanthamoeba* species are causative agents of encephalitis in humans as well as in a variety of other species including, but not limited to, baboons, monkeys, dogs, mice, and bovines [1,2]. In spite of treatment, the fatality rate for human encephalitic disease caused by free-living amoebae remains > 90% [3,4]. In addition, *Acanthamoeba* species can manifest as a cutaneous or keratitis infection; *Balamuthia mandrillaris* is also capable of manifesting as a cutaneous infection [1]. Drug discovery efforts against these amoebae have been scarce, though the encephalitic syndromes caused by them result in death the majority of the time. Further effort is warranted to identify novel therapeutics against the amoebae described below.

*Balamuthia mandrillaris* was initially isolated by Visvesvara et al. [5], in 1986, from the brain of a pregnant mandrill baboon. This protozoan parasite is presumed to occupy soil and freshwater environments and is capable of transforming into a highly resistant ternate cyst under adverse conditions [2,6,7,8]. Nonetheless, contact with soil is considered a predisposing factor for these infections, as *Balamuthia* has only been isolated from this source, making it a likely reservoir for this amoeba [8,9,10]. *B. mandrillaris* is an etiological agent for subacute or chronic *Balamuthia* amoebic encephalitis (BAE), the central nervous system (CNS) infection, as well as cutaneous and systemic infections in both animals and humans [2,11,12]. A key differentiating factor for BAE is that cases have been reported not only in immunocompromised hosts but also in immunocompetent individuals, with a higher frequency in young children and the elderly [12,13,14,15,16,17]. It is speculated that the route of entry for this parasite is either via a skin ulceration or the lower respiratory tract followed by hematogenous dissemination to the brain where the amoeba likely enters the CNS through the middle cerebral arterial supply to the choroid plexus [6,12,18].

The case fatality rate for BAE is ~92%, with death resulting within one week to several months after the initial onset of symptoms [15,19]. Clinical manifestations of BAE include drowsiness, change in mental status or behavior, low-grade fever, headache, stiff neck, hemiparesis, aphasia, cranial nerve palsies, and seizures [6,13,14]. The U.S. Centers for Disease Control and Prevention (CDC) recommends a multidrug regimen that is based on a limited number of successful clinical cases and previously identified in vitro drug susceptibility. The therapeutic drug cocktail consists of pentamidine, sulfadiazine, flucytosine, fluconazole, azithromycin, or clarithromycin, and recently, miltefosine has been added [4,19]. Despite the use of these drugs, BAE has high mortality rates and poor treatment outcomes.

*Naegleria fowleri*, the second of the three amoebae screened in this study, is the only species of the genus that is pathogenic to humans. It is the causative agent of primary amoebic meningoencephalitis (PAM), a rapidly fatal infection. First identified as a pathogenic agent in 1965, *N. fowleri* has received minimal research attention though PAM cases are > 97% fatal and highly publicized when they occur [11,20]. As a thermophilic free-living amoeba inhabiting both soil and freshwater, the number of individuals worldwide with the potential to be infected by *N. fowleri* is substantial. With global temperatures increasing, the habitable freshwater sources where *N. fowleri* can thrive have expanded as well. Typically, most PAM cases in the U.S. occur in the warmer southern-tiered states [1]. In the past 10 years, PAM cases have been reported in northern U.S. states, as far north as Minnesota, which previously had no reported cases [21]. PAM is characterized by non-specific symptoms such as headache, fever, nausea, and vomiting during the early stages of infection. Late-stage symptoms include stiff neck, confusion, hallucinations, and seizures [22]. Death typically occurs 5–12 days after initial symptom onset [11]. With non-specific early symptoms and late-stage symptoms that are identical to those of viral and bacterial meningitis, PAM is often misdiagnosed and thus likely under-reported.

Infection occurs when *N. fowleri*-contaminated water enters the nasal cavity. Most commonly, individuals are infected when swimming or playing in untreated freshwater, such as rivers and lakes, and improperly chlorinated swimming pools [23]. A significant number of cases have also been linked to neti-pot use [21]. Upon entering the nasal cavity, amoebae that traverse the cribriform plate via the olfactory nerve find their way into the frontal lobe of the brain. In the brain, *N. fowleri* causes hemorrhage and incites a massive inflammatory response that ultimately results in patient coma and death [1,11]. The standard treatment for PAM is a combinational therapeutic cocktail that includes amphotericin B, an azole (ketoconazole, fluconazole, miconazole), azithromycin, rifampin, and more recently, miltefosine [4,23]. Induced hypothermia has been tried in addition to the therapeutic cocktail with variable success [21], although mortality rates remain high with even the best available treatment regimens.

In 1930, Castellani discovered *Acanthamoeba*, another genus of free-living amoebae found in freshwater and soil, to be potentially pathogenic [18]. *Acanthamoeba*, the last of the three amoebae screened in this study, typically cause disease in immunocompromised patients but are also capable of facultatively infecting the cornea of immunocompetent contact lens wearers causing an extremely debilitating disease known as *Acanthamoeba* keratitis (AK). The symptoms of AK develop within a few days to weeks and consist of overactive tear production, photophobia, inflammation/redness, stromal infiltration, epithelial changes, edema, stromal opacity, peripheral perineural infiltrates, and incapacitating pain due to radial neuritis [24]. In immunocompromised individuals, the protist cannot not only cause cutaneous amoebiasis and nasopharyngeal infections, but it can also disseminate to the CNS causing granulomatous amoebic encephalitis (GAE) [25]. Similarly, to *B. mandrillaris*, *Acanthamoeba* is suspected to gain entry to the dead-end host via a skin lesion or the respiratory tract and then through hematogenous dissemination to the brain [26]. Clinical manifestations of GAE include headache, fever, personality alterations, somnolence, hemiparesis, aphasia, diplopia, nausea, dizziness, cranial nerve palsies, seizures, and coma [26,27]. The therapeutic measures utilized for infections caused by this parasite are laborious and ineffective, possibly due to the likelihood of inducing encystation and an inability to kill the double-walled resistant cyst stage that can be found in host tissues, unlike in PAM.

Though there are no drugs specifically approved for treatment by the U.S. Food and Drug Administration (FDA), the current treatment regimen for AK consists of a biguanide (polyhexamethylene biguanide (PHMB) or chlorhexidine) and a diamidine (propamidine or hexamidine) with hourly administration for 48 h followed by a cessation of the nighttime dosing for 2 days, concluding with 3–4 weeks of treatment every 2 h [24,28,29]. Even with this arduous treatment plan, there is the possibility for recrudescence of disease, toxic keratopathy, and vision impairment, with 2% of patients becoming blind [30]. The combinational chemotherapeutic regimen for GAE consists of an azole (ketoconazole, fluconazole, itraconazole, or voriconazole), pentamidine isethionate, sulfadiazine, amphotericin B, azithromycin, rifampin, and miltefosine [4,11,31,32,33]. This cocktail of drugs remains ineffective even with the implementation of extreme measures including surgery and cryotherapy with most cases ending in death [1,31]. Furthermore, due to the renascent nature of the cyst stage, a longer period of treatment is needed, which leads to additional trepidation for the development of drug resistance. Overall, the numbers of cases of AK have shown a significantly increasing trend worldwide in the past few decades with the estimated number of cases being up to 1.5 per 10,000 contact lens wearers depending on location [3,34,35,36,37,38,39]. Overall, the lack of favorable outcomes in BAE or GAE treatment, the continued high mortality rates for PAM, and the accretion of AK cases exemplifies the need for novel drug discovery for these deadly amoebae.

The Medicines for Malaria Venture (MMV) Pandemic Response Box is a drug library that was made available in January 2019 and is provided free of charge for research purposes. Thus far, 37 copies of this drug library have been distributed worldwide. The library consists of 400 compounds that are either already available on the market or are in various stages of drug discovery or development. It is composed of 201 (50.25%) antibacterial compounds, 153 (38.25%) antiviral compounds, and 46 (11.5%) antifungal compounds. Previous high-throughput screening efforts on free-living amoebae have yielded promising leads for therapeutic development [40,41,42,43,44,45,46,47]. However, there is still a need for the discovery and development of novel chemical scaffolds potent against these amoebae. The diverse mechanisms of action (MOAs) of the compounds found within the Pandemic Response Box represent a promising opportunity for anti-amoebic drug discovery and a starting point for structure-based drug design (SBDD).

Herein we describe the trophocidal activity of 400 bioactive compounds from screening the MMV Pandemic Response Box independently against each of the pathogenic free-living amoebae: *B. mandrillaris*, *N. fowleri*, and *A. castellanii*.

## 2. Results

### 2.1. Screening Results for Single-Point Assays

**Figure 1:**
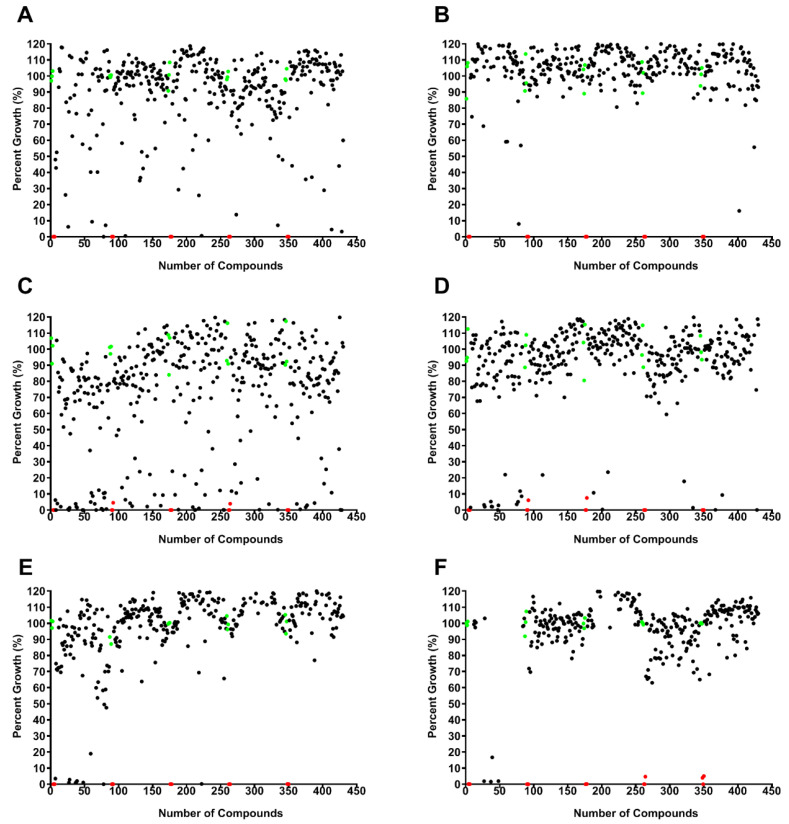
Single-point screening assay of 400 compounds from the Medicines for Malaria Venture (MMV) Pandemic Response Box were carried out using pathogenic *Balamuthia mandrillaris* at 10 µM (**A**) and 1 µM (**B**) (*n* = 1), *Naegleria fowleri* at 10 µM (**C**) and 1 µM (**D**) (*n* = 1), and *Acanthamoeba castellanii* at 10 µM (**E**) and 1 µM (**F**) (*n* = 1). Each black circle represents an individual compound response, the red circles are positive controls (chlorhexidine, 12.5 µM), and the green circles are negative growth controls (0.1% and 0.01% DMSO for 10 and 1 µM plates, respectively) included on each plate.

A library of 400 drug-like compounds, with diverse MOAs, was assembled at 10 mM in dimethyl sulfoxide (DMSO) by Medicines for Malaria Venture (MMV, Geneva, Switzerland) for open-access screening against neglected and infectious diseases. Initially, each compound was screened at two concentrations, 10 and 1 µM, against pathogenic amoebae *B. mandrillaris* (Figure 1A,B; Supplemental Figure S1)*, N. fowleri* (Figure 1C,D), and *A. castellanii* (Figure 1E,F). We used cell viability, determined with the CellTiter-Glo (CTG) 2.0 assay, as the endpoint for the single-point screening assays for all amoebae. The single-point assays yielded 43 compounds that were active (>33% inhibition) against *B. mandrillaris*, 104 compounds for *N. fowleri*, and 24 compounds for *A. castellanii*. Of the 43 compounds identified against *B. mandrillaris*, 14 compounds (33%) were antifungals, 13 compounds (30%) were antivirals, and 16 compounds (37%) were antibacterials. For *N. fowleri* we identified activity of 24 antifungal compounds (23%), 32 antivirals (31%), and 48 antibacterials (46%). Of the 24 compounds identified against *A. castellanii*, 15 compounds (62%) were antifungals, 4 compounds (17%) were antivirals, and 5 compounds (21%) were antibacterials.

### 2.2. Dose–Response Screening Results

By using the CTG assay, we performed quantitative dose–response assays to reconfirm the potency of hit compounds from single-point screens against all amoebae. All hits that produced ≥33% inhibition in the primary screen were assessed with duplicate twofold serial dilutions to determine the 50% inhibitory concentration (IC_50_). We reconfirmed 12 hits against *B. mandrillaris* (Table 1), 29 against *N. fowleri* (Table 2), and 14 against *A. castellanii* (Table 3), with potencies ranging from nanomolar to low micromolar. We counter-screened all the reconfirmed hits for cytotoxicity with the 3-(4,5-dimethylthiazol-2-yl)-5-(3-carboxymethoxyphenyl)-2-(4-sulfophenyl)-2H-tetrazolium (MTS) assay against A549 lung carcinoma cells and identified only 10 compounds that displayed cytotoxicity from <310 nM to 8 µM; all others tested were ≥ 10 µM. We determined the selectivity index (A549 IC_50_/Amoebae IC_50_), and defined an index of ≥ 10 to be our standard for further evaluation as a potentially useful drug for treatment of these parasitic diseases.

### 2.3. Comparison of Compound Activity against Three Pathogenic Amoebae 

**Figure 2:**
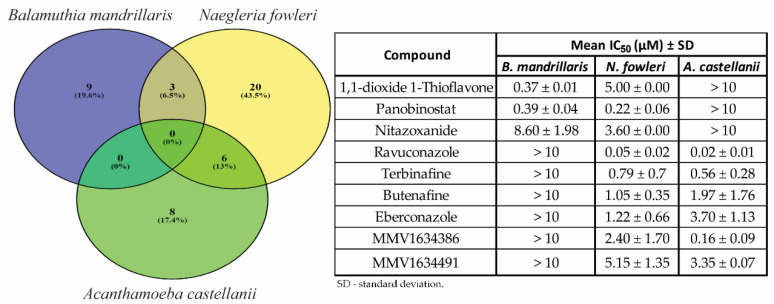
Venn diagram depicting the activity against *Balamuthia mandrillaris* (**purple**), *Naegleria fowleri* (**yellow**), and *Acanthamoeba castellanii* (**green**) of reconfirmed hits from the Medicines for Malaria Ventures (MMV) Pandemic Response Box.

We compared results for all of the reconfirmed hits against the three pathogenic amoebae to determine whether there were any compounds with potential for treating multiple pathogenic amoeba disease indications. We identified three compounds with activity that overlapped between *B. mandrillaris* and *N. fowleri* (1,1-dioxide 1-thioflavone, panobinostat, and nitazoxanide) and six compounds overlapping between *N. fowleri* and *A. castellanii* (ravuconazole, terbinafine, butenafine, eberconazole, MMV1634386, and MMV1634491) (Figure 2). We did not find *Balamuthia* and *Acanthamoeba* to share any active hits. No compounds produced > 50% at 10 µM inhibition for all three pathogenic amoebae.

## 3. Discussion

There is a great unmet medical need for new therapeutics against the diseases caused by *B. mandrillaris*, *N. fowleri*, and *Acanthamoeba* spp. These are the epitome of neglected diseases with no major pharmaceutical companies and only a few academic labs working to discover new drugs. Phenotypic screening has significantly advanced the discovery and development of drugs targeting other parasitic protozoans and has been shown to be useful for the discovery of new drugs against pathogenic free-living amoebae as well [42,43,44]. In this study, we screened 400 bioactive compounds from the MMV Pandemic Response Box for activity against all three pathogenic free-living amoebae. Given the lack of drug discovery against these pathogens, the overarching goal is to find compounds with potential for treating multiple disease indications caused by these amoebae. In addition, we aimed to discover new drugs that could be repurposed as well as new starting points for drug discovery and lead optimization. We were successful in discovering 58 new hits from this rich resource of bioactive compounds, with some having activity against at least two of the amoebae pathogens.

After reproducibly reconfirming hits via quantitative dose–response, we identified 12 compounds with IC_50_s < 10 µM against *B. mandrillaris*. Compounds with nanomolar potency included 1,1-dioxide 1-thioflavone, panobinostat, MMV1580844, MMV1582495, and MMV1634399. Pharmacological properties for 1,1-dioxide 1-thioflavone implicate its usage as an anticarcinogenic as well as an antimicrobial agent, with specific antiviral properties against cytomegalovirus and coxsackievirus [48]. Panobinostat, a histone deacetylase (HDAC) inhibitor was active against the amoeba, but it was not selective with toxicity at very low concentrations [49]. Though the molecule is approved and registered as a combinational chemotherapeutic to treat multiple myeloma, the mutagenicity and genotoxicity along with the poor pharmacokinetic profile of the drug significantly decreases the viability of the drug for amoebae, but suggests that HDAC might be a target for future studies [50]. MMV1581558, MMV021759, URMC-099-C, MMV1634394, nitazoxanide, clemizole, and selinexor all demonstrated IC_50_s ≤ 10 µM against *B. mandrillaris*. The mixed lineage kinase type 3 inhibitor, URMC-099-C, is known to induce amyloid-beta clearance in mouse Alzheimer’s models [51,52]. Though shown to be toxic, the brain-penetrating property of this molecule makes it an interesting lead compound for future drug discovery efforts. Nitazoxanide is FDA-approved and licensed to treat cryptosporidial diarrhea and other intestinal parasitic infections [53]. This thiazolide agent interferes with the electron transfer reaction in anaerobic metabolism and has been found to both inhibit Ebolavirus growth and increase the host antiviral response [54,55]. Griffin et al. [56] found that the benzimidazole histamine H1 antagonist, clemizole, acts as an antiepileptic and speculate that it could be used to treat Dravet syndrome based on their study in zebrafish. Clemizole has also been implicated as a potential therapeutic against hepatitis C and shows antitumor as well as anti-allergic activities [57]. Selinexor acts by inhibiting the nuclear export protein, exportin 1, and has received FDA approval for the treatment of multiple myeloma in the U.S. [58].

In total, 29 compounds reproducibly yielded IC_50_ values ≤ 10 µM for *N. fowleri*. The majority (22 of 29) of the hit compounds have not been previously reported in the literature for activity against *N. fowleri*. The Pandemic Response Box screen yielded seven active compounds with nanomolar potency against *N. fowleri*: luliconazole, ravuconazole, CRS-3123, fludarabine, panobinostat, erythromycin, and terbinafine. Aside from fludarabine and panobinostat, these compounds all generated selectivity indices ≥ 10, preliminarily indicating little cytotoxicity to A549 mammalian cells. Luliconazole and ravuconazole are novel compounds, whose activity has not been previously described against *N. fowleri*. Luliconazole is an FDA-approved imidazole antifungal agent that is typically administered in a topical cream, and primarily prescribed for the fungal foot infection, tinea pedis [59]. Currently, luliconazole is being investigated for its use against a broad spectrum of fungal afflictions including aspergillosis, dermatophytosis, and onychomycosis [60]. Ravuconazole is a triazole antifungal approved for clinical use in Japan for treatment of tinea pedis but has yet to receive FDA approval [61]. In vivo studies have demonstrated promising results for oral and intravenous bolus administration of ravuconazole. Groll et al. [62] have shown ravuconazole is able to penetrate the blood–brain barrier (BBB) and reach brain tissue in an in vivo rabbit study. Ravuconazole has also been effective in vivo in models for disseminated candidiasis, intracranial cryptococcosis, and invasive pulmonary aspergillosis [63,64,65]. Eberconazole was also identified as a hit compound against *N. fowleri*, making this the first report of its activity against this parasite. Eberconazole is an imidazole antifungal that is currently not FDA approved. Eberconazole has shown promising in vitro results for dermatophytosis and candidiasis [66].

Luliconazole, ravuconazole, and eberconazole function by blocking ergosterol biosynthesis via inhibition of 14α-demethylase [67]. The azole class of drugs has long been known to be active against *N. fowleri* and an azole has been included in the treatment regimen since the 1980s [68]. Furthermore, 14 α-demethylase has been confirmed as a drug target against *N. fowleri* [69]. Posaconazole is the most recently described azole with promise as a therapeutic lead and is implicated as an effective combinational partner in the treatment for PAM [42]. Both luliconazole and ravuconazole produced lower IC_50_ values than the reported posaconazole IC_50_, thus, these compounds warrant further in vitro and in vivo evaluation.

CRS-3123, fludarabine, panobinostat, erythromycin, and terbinafine were also identified as nanomolar inhibitors of *N. fowleri*. We rediscovered these compounds that have been described previously in high-throughput screens [42,44]. CRS-3123 is an antibacterial compound that targets methionyl tRNA-synthetase and is currently involved in clinical trials for the treatment of *Clostridium difficile* [70]. Fludarabine is a purine nucleoside analog antineoplastic with FDA approval that is used in the treatment of leukemia and lymphoma [71]. Panobinostat is an FDA-approved histone deacetylase inhibitor antineoplastic used in the treatment of multiple myeloma [49]. Erythromycin is an FDA-approved antibacterial macrolide with a broad spectrum of clinical applications [72]. Terbinafine is an amine antifungal that inhibits squalene epoxidase and also is FDA approved [73]. Though not nanomolar hits, butenafine and repatamulin also were reconfirmed as hit compounds ≤ 10 µM in this screen. Reconfirmation of hits identified from previous screening demonstrates the robustness of our assay and the effectiveness of our high-throughput screening methods.

For *A. castellanii*, 14 compounds yielded reproducible IC_50_s ≤ 10 µM. Of the hit compounds, 9 of 14 have not been previously described in the literature for activity against *Acanthamoeba* species. Our screen identified five inhibitors with nanomolar potency against *A*. *castellanii*: ravuconazole, isavuconazonium, MMV1634386, terbinafine, and MMV1582496. Additionally, all five compounds presented a selectivity index ≥ 10 and, thus, do not appear to display cytotoxic effects against A549 mammalian cells in vitro. Of these five compounds, the three with known MOAs target ergosterol biosynthesis, with ravuconazole and isavuconazonium specifically inhibiting CYP51, and terbinafine inhibiting squalene 2,3-epioxidase [74,75,76]. Ravuconazole, an inhibitor we discovered for *N. fowleri*, also is effective at inhibiting growth of a clinical isolate of fungal keratitis caused by *Scedosporium apiospermum* and, thus, could have valuable potential in treating ocular infections caused by *Acanthamoeba* species [77]. Isavuconazonium, the pro-drug of isavuconazole, has also been shown to penetrate the BBB and is approved by the FDA to treat aspergillosis as well as mucormycosis [78,79]. The allylamine antifungal, terbinafine, has been used previously to treat a case of osteo-cutaneous acanthamoebiasis [80]. Compounds with IC_50_s ≤ 10 µM against *A. castellanii* included ketoconazole, amorolfine, trifluoroacetic acid, butenafine, alexidine, MMV1634491, eberconazole, furvina, and MMV1782221. Ketoconazole, amorolfine, butenafine, and eberconazole interfere with the ergosterol biosynthesis pathway by inhibiting CYP51A1, Δ7,8-isomerase and the C14-reductase, squalene epoxidase, as well as 14α-demethylase, respectively [81,82,83]. Although not approved in the U.S. or Canada, amorolfine is approved and commonly used topically to treat dermatophyte infections (tinea capitis, tinea pedis, and onchomycosis) in Australia and the United Kingdom [84,85]. The benzylamine antifungal, butenafine, is approved by the FDA for topical treatment of tinea pedis and has also been found to be effective against *Leishmania* and a variety of ocular pathogenic fungal infections [86,87,88]. The activity of trifluoroacetic acid—a strong carboxylic acid that is commonly utilized as a solvent and an ion pairing agent in organic reactions—presumably stems from the decrease in pH to a level that is toxic to the amoebae [89]. Alexidine, being a bis-biguanide, acts via phase separation as well as the interruption of domain formation in membrane lipids and has been shown to have activity against several *Acanthamoeba* species [90]. The synthetic nitrovinylfuran broad spectrum antibiotic, furvina, was developed in Cuba and has shown antimicrobial activity against bacteria, fungi, and yeasts by preferentially inhibiting protein synthesis at the P-site of the 30s ribosomal subunit [91]. We found ketoconazole, terbinafine, and alexidine to have reconfirmed its activity against *Acanthamoeba* as well as isavuconazonium and butenafine, both identified in a previous drug susceptibility screen [44,92,93,94]. These hits against *Acanthamoeba* further substantiate the robust nature of our high-throughput drug susceptibility screening techniques.

Unfortunately, no compounds had shared activity between all three amoebae at the final screening concentration tested of ≤ 10 µM. This could be due to our stringent activity criteria implemented to detect moderately active molecules that directly target the amoebae. Our screen would not identify immune modulators or compounds that target host processes that could affect amoeba infections. The results of this study as well as a large screen of 12,000 compounds suggest it is unlikely a potent compound (<1 µM) with pan-activity against the three-amoebae will be found without undergoing several rounds of structure–activity relationship (SAR) medicinal chemistry optimization. We did however identify three compounds that were shared between *B. mandrillaris* and *N. fowleri* (1,1-dioxide 1-thioflavone, panobinostat, and nitazoxanide) and six compounds that were shared between *N. fowleri* and *A. castellanii* (ravuconazole, terbinafine, butenafine, eberconazole, MMV1634386, and MMV1634491). Of these, ravuconazole, terbinafine, butenafine, eberconazole for *N. fowleri* and *A. castellanii* and panobinostat and nitazoxanide for *B. mandrillaris* and *N. fowleri* should be investigated further for in vivo efficacy and the potential off-label use for the various clinical diseases caused by these amoebae. In addition to the potential for repurposing, the hits identified in this study offer opportunities to confirm targets and mechanism(s) of action that will enhance our understanding of the biology of these amoebae and potentially identify new pathways for therapeutic development.

## 4. Materials and Methods

### 4.1. Maintenance of Amoebae

#### 4.1.1. Balamuthia mandrillaris

Pathogenic *Balamuthia mandrillari*s (CDC:V039; American Type Culture Collection (ATCC) 50209), a GAE isolate, isolated from a pregnant baboon at the San Diego Zoo in 1986 was donated by Luis Fernando Lares-Jiménez ITSON University, Mexico [43]. Trophozoites were routinely grown axenically in *Balamuthia mandrillari*s Itson (BMI) media at 37 °C, 5% CO_2_ in vented 75 cm^2^ tissue culture flasks (Olympus, El Cajon, CA, USA), until the cells were 80–90% confluent. For sub-culturing, 0.25% Trypsin-EDTA (Gibco, Gaithersburg, MD, USA) cell detachment reagent was used to detach the cells from the culture flasks. The cells were collected by centrifugation at 4000 rpm at 4 °C. Complete BMI media is produced by the addition of 10% fetal bovine serum (FBS) and 125 μg of penicillin/streptomycin antibiotics.

#### 4.1.2. Naegleria fowleri

Pathogenic *Naegleria fowleri* (ATCC 30215), a clinical isolate obtained from a 9-year-old boy in Adelaide, Australia, that died of PAM in 1969 was previously purchased from the American Type Culture Collection (ATCC) [40]. Trophozoites were routinely grown axenically at 34 °C in Nelson’s complete medium (NCM) in non-vented 75 cm^2^ tissue culture flasks (Olympus, El Cajon, CA, USA), until the cells were 80–90% confluent. For sub-culturing, cells were placed on ice to detach the cells from the culture flasks. The cells were collected by centrifugation at 4000 rpm at 4 °C. Complete NCM media is produced by the addition of 10% FBS and 125 μg of penicillin/streptomycin antibiotics. 

#### 4.1.3. Acanthamoeba castellanii

Pathogenic *Acanthamoeba castellanii* T4 isolate (ATCC 50370) used in these studies was isolated from the eye of a patient in New York, NY, in 1978. This isolate was also purchased from ATCC. Trophozoites were routinely grown axenically at 27 °C in Protease Peptone-Glucose Media (PG) in non-vented 75 cm^2^ tissue culture flasks (Olympus, El Cajon, CA, USA), until the cells were 80–90% confluent. For sub-culturing, cells were mechanically harvested to detach the cells from the culture flasks [95]. The cells were collected by centrifugation at 4000 rpm at 4 °C. Complete PG media is produced by the addition of 125 μg of penicillin/streptomycin antibiotics.

All experiments were performed using logarithmic phase trophozoites.

### 4.2. Compound Library

The Pandemic Response Box (https://www.mmv.org/mmv-open/pandemic-response-box) was modeled after the previously successful Malaria and Pathogen Boxes and is an open-source drug library that consists of 400 diverse compounds that include: 201 antibacterial inhibitors (50.25%), 153 antiviral inhibitors (38.25%), and 46 antifungal inhibitors (11.5%). Compounds within this collection are either FDA approved and currently available on the pharmaceutical market or in earlier different stages of drug development. All stock compounds were supplied as 10 mM in DMSO. 

### 4.3. In Vitro CellTiter-Glo Trophocidal Assay 

The trophocidal activity of compounds was assessed using the CellTiter-Glo 2.0 luminescent viability assay (Promega, Madison, WI, USA), as previously described [40,43]. Trophozoites were routinely cultured as described above and only logarithmic trophozoites were used. In brief, *B. mandrillaris*, *N. fowleri*, or *A. castellanii* trophozoites cultured in their corresponding media were seeded at 16,000, 3000, or 1440 cells/well into white 96-well plates (Costar 3370), respectively. Initially all compounds were assessed in a single-point drug screen at 10 and 1 μM, as previously described. Inhibitors were assessed for percent inhibition using the criteria of ≤33% growth inhibition (no inhibition), 33–67% growth inhibition (moderate inhibition), and ≥67% growth inhibition (strong inhibition). Control wells were supplemented with 0.1% (10 μM screening plate) or 0.01% (1 μM screening plate) DMSO, as the negative controls, or 12.5 μM of chlorhexidine, as the positive control. Following the single-point assays at 10 and 1 µM, we prioritized compounds for progression to IC_50_ determinations. Quantitative dose–response assays were conducted with compounds that inhibited parasite growth by 50% or greater at the 10 µM concentration and the same compound inhibited by >33% at 1 µM. We used this process to reduce the number of potential false positives. Drugs were cherry-picked, dissolved in the media specific to each parasite, and assessed in twofold serial dilutions from the highest concentration of 10 μM. All assays were incubated at each of the parasites’ representative growth temperatures, described above, for 72 h. At the 72 h time point, 25 μL of CellTiter-Glo 2.0 reagent was added to all wells of the 96-well plates using the Biomek NX^P^ automated workstation. The plates were protected from light and contents were mixed using an orbital shaker at 300 rpm at room temperature for 2 min to induce cell lysis. After shaking, the plates were equilibrated at room temperature for 10 min to stabilize the luminescent signal. The adenosine triphosphate (ATP) luminescent signal (relative light units; RLUs) was measured at 490 nm with a SpectraMax I3X plate reader (Molecular Devices, Sunnyvale, CA, USA). Drug inhibitory concentration (IC_50_) curves were generated using total ATP RLUs, where controls were calculated as the average of replicates using the Levenberg–Marquardt algorithm, using DMSO as the normalization control, as defined in Collaborative Drug Discovery Inc. (CDD) Vault (Burlingame, CA, USA). Values reported are from a minimum of two biological replicates with standard deviations.

### 4.4. Cytotoxicity Screening of Reconfirmed Hits

Cytotoxicity of the reconfirmed hits was determined by using the CellTiter 96^®^ AQ_ueous_ One Solution Cell Proliferation Assay (Promega, Madison, WI) on A549 human lung carcinoma cells [40]. A549 cells were seeded at a concentration of 1.6 × 10^4^ cells/mL in 96-well tissue culture plates (Corning, NY, USA), in the presence of serially diluted active hits against *B. mandrillaris, N. fowleri*, or *A. castellanii*. Positive control wells contained cells and media; negative control wells contained 0.1% DMSO. Cells were grown in F12K medium supplemented with 10% FBS and 1% gentamycin (all supplied from Fisher Scientific, Atlanta, GA, USA). The inhibitor concentration started at 10 µM and was diluted in doubling dilutions to assess cytotoxicity in comparison to the respective free-living amoeba. Total volume of each well was 100 µL and plates were incubated at 37 °C, 5% CO_2_ for 72 h. Next, 4 h before the time-point, 20 µL of MTS (Promega, Madison, WI, USA) was added to each well. Inhibition of A549 growth was assessed at the 72 h time-point measuring the optical density (OD) values at 490 nm using a SpectraMax I3X plate reader (Molecular Devices, Sunnyvale, CA, USA). Curve fitting using non-linear regression was carried out using the average of replicates and the Levenberg–Marquardt algorithm, using DMSO as the normalization control, as defined in CDD Vault (Burlingame, CA, USA).

From these data we calculated a selectivity index (SI), SI = (IC_50_ A549)/(IC_50_ Amoeba). An SI value ≥ 10 was considered the standard for further evaluation as a potentially useful drug. 

### 4.5. Statistical Analysis

We used the Z’ factor as a statistical measurement to assess the robustness of our high-throughput screening assays (Figure 1) [96]. This factor uses the mean and standard deviation values of the positive and negative controls to assess data quality. The robustness of all of the plates screened had an excellent Z’-score value of 0.8 or above. 

## 5. Conclusions

From a library of 391 compounds screened for the first time against *B. mandrillaris*, *N. fowleri*, or *A. castellanii,* we identified multiple new hits with nanomolar to low micromolar potency against one or more of the pathogens. A few of these drugs have been used clinically for other indications and represent potential repurposing candiates, whereas other inhibitors can be evaluated to identify mechanism(s) of action, as starting points for medicinal chemistry hit optimization, and new targets for chemotherapy for these pathogenic free-living amoebae. 

## Figures and Tables

**Table 1:** Dose–response activity of confirmed hits against *B. mandrillaris*.

Compound Name	*B. mandrillaris* Mean IC_50_ (µM) ± SD	A549 Mean IC_50_ (µM)	Selectivity Index
1,1-dioxide 1-thioflavone	0.37 ± 0.01	>10	≥27.40
Panobinostat	0.39 ± 0.04	<0.31	<0.79
MMV1580844	0.53 ± 0.11	>10	≥19.10
MMV1582495	0.54 ± 0.04	>10	≥18.50
MMV1634399	0.73 ± 0.28	>10	≥13.70
MMV1581558	2.00 ± 0.99	>10	≥5.00
MMV021759	4.50 ± 0.42	>10	≥2.22
URMC-099-C	5.35 ± 1.63	4.2	0.79
MMV1634394	8.05 ± 2.19	>10	≥1.24
Nitazoxanide	8.60 ± 1.98	>10	≥1.16
Clemizole	8.95 ± 1.48	>10	≥1.12
Selinexor	9.20 ± 1.13	>10	≥1.09

Inhibitory concentration determined by the CellTiter-Glo (CTG) method in duplicate and values of 50% inhibitory concentration (IC_50_) expressed as mean ± standard deviation (SD).

**Table 2:** Dose–response activity of confirmed hits against *N. fowleri*.

Compound Name	*N. fowleri* Mean IC_50_ (µM) ± SD	A549 Mean IC_50_ (µM)	Selectivity Index
Luliconazole	0.02 ± 0.01	>10	≥556
Ravuconazole	0.05 ± 0.02	>10	≥250
MMV1578884	0.08 ± 0.02	>10	≥128
Fludarabine	0.11 ± 0.03	<0.31	<2.83
Panobinostat	0.22 ± 0.06	<0.31	<1.44
Erythromycin	0.24 ± 0.02	>10	≥42.6
Terbinafine	0.79 ± 0.70	>10	≥12.7
Butenafine	1.05 ± 0.35	>10	≥9.52
Retapamulin	1.12 ± 0.28	>10	≥8.97
Eberconazole	1.22 ± 0.66	>10	≥8.23
Tipifarnib	1.23 ± 0.27	8.00	6.50
Rubitecan	2.30 ± 0.20	0.47	0.20
MMV1634386	2.40 ± 1.70	>10	≥4.18
Epetraborole	2.60 ± 0.30	>10	≥3.85
Eravacycline	2.75 ± 0.35	>10	≥3.64
Nitazoxanide	3.60 ± 0.00	>10	≥2.78
Triapine	4.00 ± 0.10	3.50	0.88
MMV1580853	4.95 ± 2.55	>10	≥2.02
1,1-dioxide 1-thioflavone	5.00 ± 0.00	>10	≥2.00
MMV1634491	5.15 ± 1.35	>10	≥1.94
MMV1593541	5.30 ± 0.60	2.30	0.43
RWJ-67657	5.35 ± 0.35	>10	≥1.87
MMV1782222	5.40 ± 0.00	>10	≥1.85
MMV019724	6.00 ± 0.70	6.20	1.03
MMV1578570	6.40 ± 0.90	>10	≥1.56
Ciclopirox	7.50 ± 1.40	>10	≥1.33
MMV1782214	7.90 ± 0.00	>10	≥1.27
MMV1633967	8.20 ± 1.00	>10	≥1.22
MMV1782115	9.35 ± 0.35	5.80	0.62

Inhibitory concentration determined by the CTG method in duplicate and values of IC_50_ expressed as mean ± standard deviation (SD).

**Table 3:** Dose–response activity of confirmed hits against *A. castellanii*.

Compound Name	*A. castellanii* Mean IC_50_ (µM) ± SD	A549 Mean IC_50_ (µM)	Selectivity Index
Ravuconazole	0.02 ± 0.01	>10	≥625
Isavuconazonium	0.09 ± 0.02	>10	≥105
MMV1634386	0.16 ± 0.09	>10	≥62.50
Terbinafine	0.56 ± 0.28	>10	≥18
MMV1582496	0.61 ± 0.35	>10	≥16.40
Ketoconazole	1.24 ± 0.80	>10	≥8.10
Amorolfine	1.63 ± 1.62	>10	≥6.13
Trifluoroacetic acid	1.97 ± 1.00	>10	≥5.10
Butenafine	1.97 ± 1.76	>10	≥5.08
Alexidine	2.40 ± 1.56	2.10	0.88
MMV1634491	3.35 ± 0.07	>10	≥2.99
Eberconazole	3.70 ± 1.13	>10	≥2.70
Furvina	4.20 ± 0.85	>10	≥2.38
MMV1782221	4.43 ± 1.00	>10	≥2.26

Inhibitory concentration determined by the CTG method in duplicate and values of IC_50_ expressed as mean ± standard deviation (SD).

## Supplementary Materials

The following are available online at https://www.mdpi.com/2076-0817/9/6/476/s1, Figure S1. Morphology and density of *Balamuthia mandrillaris* trophozoites in a negative control well after 72 hr (16,000 amoebae/well).

## References

[1] Schuster F.L., Visvesvara G.S. (2004). Opportunistic Amoebae: Challenges in Prophylaxis and Treatment. Drug Resist. Updat..

[2] Visvesvara G.S., Schuster F.L., Martinez A.J. (1993). Balamuthia Mandrillaris, N. G., N. Sp., Agent of Amebic Meningoencephalitis In Humans and Other Animals. J. Eukaryot. Microbiol..

[3] Trabelsi H., Dendana F., Sellami A., Sellami H., Cheikhrouhou F., Neji S., Makni F., Ayadi A. (2012). Pathogenic Free-Living Amoebae: Epidemiology and Clinical Review. Pathol. Biol..

[4] Cope J.R. (2013). Investigational Drug Available Directly from CDC for the Treatment of Infections with Free-Living Amebae. Morb. Mortal. Wkly. Rep..

[5] Visvesvara G.S., Martinez A.J., Schuster F.L., Leitch G.J., Wallace S.V., Sawyer T.K., Anderson M. (1990). Leptomyxid Ameba, a New Agent of Amebic Meningoencephalitis in Humans and Animals. J. Clin. Microbiol..

[6] Martinez A.J., Schuster F.L., Visvesvara G.S. (2001). Balamuthia Mandrillaris: Its Pathogenic Potential. J. Eukaryot. Microbiol..

[7] Siddiqui R., Matin A., Warhurst D., Stins M., Khan N.A. (2007). Effect of Antimicrobial Compounds on Balamuthia Mandrillaris Encystment and Human Brain Microvascular Endothelial Cell Cytopathogenicity. Antimicrob. Agents Chemother..

[8] Lorenzo-Morales J., Cabello-Vílchez A.M., Martín-Navarro C.M., Martínez-Carretero E., Piñero J.E., Valladares B. (2013). Is Balamuthia Mandrillaris a Public Health Concern Worldwide?. Trends Parasitol..

[9] Bravo F.G., Alvarez P.J., Gotuzzo E. (2011). Balamuthia Mandrillaris Infection of the Skin and Central Nervous System: An Emerging Disease of Concern to Many Specialties in Medicine. Curr. Opin. Infect. Dis..

[10] Niyyati M., Lorenzo-Morales J., Rezaeian M., Martin-Navarro C.M., Haghi A.M., MacIver S.K., Valladares B. (2009). Isolation of Balamuthia Mandrillaris from Urban Dust, Free of Known Infectious Involvement. Parasitol. Res..

[11] Visvesvara G.S., Moura H., Schuster F.L. (2007). Pathogenic and Opportunistic Free-Living Amoebae: *Acanthamoeba* Spp., Balamuthia Mandrillaris, Naegleria Fowleri, and Sappinia Diploidea. FEMS Immunol. Med. Microbiol..

[12] Deol I., Robledo L., Meza A., Visvesvara G.S., Andrews R.J. (2000). Encephalitis Due to a Free-Living Amoeba (Balamuthia Mandrillaris): Case Report with Literature Review. Surg. Neurol..

[13] Takei K., Toyoshima M., Nakamura M., Sato M., Shimizu H., Inoue C., Shimizu Y., Yagita K. (2018). An Acute Case of Granulomatous Amoebic Encephalitis-Balamuthia Mandrillaris Infection. Intern. Med..

[14] Denney C.F., Iragui V.J., Uber-Zak L.D., Karpinski N.C., Ziegler E.J., Visvesvara G.S., Reed S.L. (1997). Amebic Meningoencephalitis Caused by Balamuthia Mandrillaris: Case Report and Review. Clin. Infect. Dis..

[15] Krasaelap A., Prechawit S., Chansaenroj J., Punyahotra P., Puthanakit T., Chomtho K., Shuangshoti S., Amornfa J., Poovorawan Y. (2013). Fatal Balamuthia Amebic Encephalitis in a Healthy Child: A Case Report with Review of Survival Cases. Korean J. Parasitol..

[16] Deetz T.R., Sawyer M.H., Billman G., Schuster F.L., Visvesvara G.S. (2003). Successful Treatment of Balamuthia Amoebic Encephalitis: Presentation of 2 Cases. Clin. Infect. Dis..

[17] Shehab K.W., Aboul-Nasr K., Elliott S.P. (2018). Balamuthia Mandrillaris Granulomatous Amebic Encephalitis with Renal Dissemination in a Previously Healthy Child: Case Report and Review of the Pediatric Literature. J. Pediatric Infect. Dis. Soc..

[18] Ong T.Y.Y., Khan N.A., Siddiqui R. (2017). Brain-Eating Amoebae: Predilection Sites in the Brain and Disease Outcome. J. Clin. Microbiol..

[19] Cope J.R., Landa J., Nethercut H., Collier S.A., Glaser C., Moser M., Puttagunta R., Yoder J.S., Ali I.K., Roy S.L. (2019). The Epidemiology and Clinical Features of Balamuthia Mandrillaris Disease in the United States, 1974–2016. Clin. Infect. Dis..

[20] Fowler M., Carter R. (1965). Female Urethra-Ferris of Female Acute Pyogenic Meningitis Probably Due. Br. Med. J..

[21] Cope J.R., Ali I.K. (2016). Primary Amebic Meningoencephalitis: What Have We Learned in the Last 5 Years?. Curr. Infect. Dis. Rep..

[22] Capewell L.G., Harris A.M., Yoder J.S., Cope J.R., Eddy B.A., Roy S.L., Visvesvara G.S., Fox L.A.M., Beach M.J. (2015). Diagnosis, Clinical Course, and Treatment of Primary Amoebic Meningoencephalitis in the United States, 1937–2013. J. Pediatric Infect. Dis. Soc..

[23] Bellini N.K., Santos T.M., da Silva M.T.A., Thiemann O.H. (2018). The Therapeutic Strategies against Naegleria Fowleri. Exp. Parasitol..

[24] Szentmáry N., Daas L., Shi L., Laurik K.L., Lepper S., Milioti G., Seitz B. (2019). Acanthamoeba Keratitis—Clinical Signs, Differential Diagnosis and Treatment. J. Curr. Ophthalmol..

[25] Martinez A.J., Janitschke K. (1985). Acanthamoeba, an opportunistic microorganism: A review. Infection.

[26] Siddiqui R., Khan N.A. (2012). Biology and Pathogenesis of Acanthamoeba. Parasit. Vectors.

[27] Martinez A.J. (1991). Infection of the Central Nervous System Due to Acanthamoeba. Rev. Infect. Dis..

[28] Maycock N.J.R., Jayaswal R. (2016). Update on Acanthamoeba Keratitis: Diagnosis, Treatment, and Outcomes. Cornea.

[29] Khan N.A. (2006). Acanthamoeba: Biology and Increasing Importance in Human Health. FEMS Microbiol. Rev..

[30] Dart J.K.G., Saw V.P.J., Kilvington S. (2009). Acanthamoeba Keratitis: Diagnosis and Treatment Update 2009. Am. J. Ophthalmol..

[31] Visvesvara G.S. (2010). Amebic Meningoencephalitides and Keratitis: Challenges in Diagnosis and Treatment. Curr. Opin. Infect. Dis..

[32] Aichelburg A.C., Walochnik J., Assadian O., Prosch H., Steuer A., Perneczky G., Visvesvara G.S., Aspöck H., Vetter N. (2008). Successful Treatment of Disseminated Acanthamoeba Sp. Infection with Miltefosine. Emerg. Infect. Dis..

[33] Schuster F.L., Guglielmo B.J., Visvesvara G.S. (2006). In-Vitro Activity of Miltefosine and Voriconazole on Clinical Isolates of Free-Living Amebas: Balamuthia Mandrillaris, Acanthamoeba Spp., and Naegleria Fowleri. J. Eukaryot. Microbiol..

[34] Khan N.A., Anwar A., Siddiqui R. (2018). Acanthamoeba Keratitis: Current Status and Urgent Research Priorities. Curr. Med. Chem..

[35] Seal D.V. (2003). Acanthamoeba Keratitis Update—Incidence, Molecular Epidemiology and New Drugs for Treatment. Eye.

[36] Carvalho F.R.S., Foronda A.S., Mannis M.J., Höfling-Lima A.L., Belfort R., De Freitas D. (2009). Twenty Years of Acanthamoeba Keratitis. Cornea.

[37] Gatti S., Rama P., Matuska S., Berrilli F., Cavallero A., Carletti S., Bruno A., Maserati R., Di Cave D. (2010). Isolation and Genotyping of Acanthamoeba Strains from Corneal Infections in Italy. J. Med. Microbiol..

[38] Verani J.R., Lorick S.A., Yoder J.S., Beach M.J., Braden C.R., Roberts J.M., Conover C.S., Chen S., McConnell K.A., Chang D.C. (2009). National Outbreak of Acanthamoeba Keratitis Associated with Use of a Contact Lens Solution, United States. Emerg. Infect. Dis..

[39] Carnt N., Hoffman J.J., Verma S., Hau S., Radford C.F., Minassian D.C., Dart J.K.G. (2018). Acanthamoeba Keratitis: Confirmation of the UK Outbreak and a Prospective Case-Control Study Identifying Contributing Risk Factors. Br. J. Ophthalmol..

[40] Rice C.A., Colon B.L., Alp M., Göker H., Boykin D.W., Kyle D.E. (2015). Bis-Benzimidazole Hits against Naegleria Fowleri Discovered with New High-Throughput Screens. Antimicrob. Agents Chemother..

[41] Van Voorhis W.C., Adams J.H., Adelfio R., Ahyong V., Akabas M.H., Alano P., Alday A., Alemán Resto Y., Alsibaee A., Alzualde A. (2016). Open Source Drug Discovery with the Malaria Box Compound Collection for Neglected Diseases and Beyond. PLoS Pathog..

[42] Colon B.L., Rice C.A., Guy R.K., Kyle D.E. (2019). Phenotypic Screens Reveal Posaconazole as a Rapidly Acting Amebicidal Combination Partner for Treatment of Primary Amoebic Meningoencephalitis. J. Infect. Dis..

[43] Rice C.A., Lares-Jiménez L.F., Lares-Villa F., Kyle D.E. (2020). In Vitro Screening of the Open Source MMV Malaria and Pathogen Boxes to Discover Novel Compounds with Activity against Balamuthia Mandrillaris. Antimicrob. Agents Chemother..

[44] Rice C.A., Colon B.L., Chen E., Hull M.V., Kyle D.E. (2020). Discovery of repurposing drug candidates for the treatment of diseases caused by pathogenic free-living amoebae. bioRxiv.

[45] Sifaoui I., Reyes-Batlle M., López-Arencibia A., Chiboub O., Bethencourt-Estrella C.J., San Nicolás-Hernández D., Rodríguez Expósito R.L., Rizo-Liendo A., Piñero J.E., Lorenzo-Morales J. (2019). Screening of the Pathogen Box for the Identification of Anti-Acanthamoeba Agents. Exp. Parasitol..

[46] Kangussu-Marcolino M.M., Ehrenkaufer G.M., Chen E., Debnath A., Singh U. (2019). Identification of Plicamycin, TG02, Panobinostat, Lestaurtinib, and GDC-0084 as Promising Compounds for the Treatment of Central Nervous System Infections Caused by the Free-Living Amebae Naegleria, Acanthamoeba and Balamuthia. Int. J. Parasitol. Drugs Drug Resist..

[47] Laurie M.T., White C.V., Retallack H., Wu W., Moser M.S., Sakanari J.A., Ang K., Wilson C., Arkin M.R., DeRisi J.L. (2018). Functional Assessment of 2,177 U.S. and International Drugs Identifies the Quinoline Nitroxoline as a Potent Amoebicidal Agent against the Pathogen Balamuthia mandrillaris. mBio.

[48] Dong J., Zhang Q., Meng Q., Wang Z., Li S., Cui J. (2018). The Chemistry and Biological Effects of Thioflavones. Mini Rev. Med. Chem..

[49] Moore D. (2016). Panobinostat (Farydak): A Novel Option for the Treatment of Relapsed or Relapsed and Refractory Multiple Myeloma. Pharm. Ther..

[50] Van Veggel M., Westerman E., Hamberg P. (2018). Clinical Pharmacokinetics and Pharmacodynamics of Panobinostat. Clin. Pharmacokinet..

[51] Marker D.F., Tremblay M.È., Puccini J.M., Barbieri J., Gantz Marker M.A., Loweth C.J., Chris Muly E., Lu S.M., Goodfellow V.S., Dewhurst S. (2013). The New Small-Molecule Mixed-Lineage Kinase 3 Inhibitor URMC-099 Is Neuroprotective and Anti-Inflammatory in Models of Human Immunodeficiency Virus-Associated Neurocognitive Disorders. J. Neurosci..

[52] Kiyota T., Machhi J., Lu Y., Dyavarshetty B., Nemati M., Zhang G., Lee Mosley R., Gelbard H.A., Gendelman H.E. (2018). URMC-099 Facilitates Amyloid-β Clearance in a Murine Model of Alzheimer’s Disease. J. Neuroinflammation..

[53] Fox L.M., Saravolatz L.D. (2005). Nitazoxanide: A New Thiazolide Antiparasitic Agent. Clin. Infect. Dis..

[54] Jasenosky L.D., Cadena C., Mire C.E., Borisevich V., Haridas V., Ranjbar S., Nambu A., Bavari S., Soloveva V., Sadukhan S. (2019). The FDA-Approved Oral Drug Nitazoxanide Amplifies Host Antiviral Responses and Inhibits Ebola Virus. iScience.

[55] Thurston S., Hite G.L., Petry A.N., Ray S.D. (2015). Antiprotozoal Drugs.

[56] Griffin A., Hamling K.R., Knupp K., Hong S.G., Lee L.P., Baraban S.C. (2017). Clemizole and Modulators of Serotonin Signalling Suppress Seizures in Dravet Syndrome. Brain.

[57] Einav S., Gerber D., Bryson P.D., Sklan E.H., Elazar M., Maerkl S.J., Glenn J.S., Quake S.R. (2008). Discovery of a Hepatitis C Target and Its Pharmacological Inhibitors by Microfluidic Affinity Analysis. Nat. Biotechnol..

[58] Syed Y.Y. (2019). Selinexor: First Global Approval. Drugs.

[59] Gupta A.K., Foley K.A., Versteeg S.G. (2017). New Antifungal Agents and New Formulations Against Dermatophytes. Mycopathologia.

[60] Hivary S., Fatahinia M., Halvaeezadeh M., Mahmoudabadi A.Z. (2019). The Potency of Luliconazole against Clinical and Environmental Aspergillus Nigri Complex. Iran. J. Microbiol..

[61] Kano R., Sugita T., Kamata H. (2020). Antifungal Susceptibility of Clinical Isolates and Artificially Produced Multi-Azole-Resistant Strains of Cryptococcus Neoformans (Formerly: Cryptococcus Grubii)to Ravuconazole. Med. Mycol. J..

[62] Groll A.H., Mickiene D., Petraitis V., Petraitiene R., Kelaher A., Sarafandi A., Wuerthwein G., Bacher J., Walsh T.J. (2005). Compartmental Pharmacokinetics and Tissue Distribution of the Antifungal Triazole Ravuconazole Following Intravenous Administration of Its Di-Lysine Phosphoester Prodrug (BMS-379224) in Rabbits. J. Antimicrob. Chemother..

[63] Hata K., Kimura J., Miki H., Toyosawa T., Moriyama M., Katsu K. (1996). Efficacy of ER-30346, a Novel Oral Triazole Antifungal Agent, in Experimental Models of Aspergillosis, Candidiasis, and Cryptococcosis. Antimicrob. Agents Chemother..

[64] Andes D., Marchillo K., Stamstad T., Conklin R. (2003). In Vivo Pharmacodynamics of a New Triazole, Ravuconazole, in a Murine Candidiasis Model. Antimicrob. Agents Chemother..

[65] Petraitiene R., Petraitis V., Lyman C.A., Groll A.H., Mickiene D., Peter J., Bacher J., Roussillon K., Hemmings M., Armstrong D. (2004). Efficacy, Safety, and Plasma Pharmacokinetics of Escalating Dosages of Intravenously Administered Ravuconazole Lysine Phosphoester for Treatment of Experimental Pulmonary Aspergillosis in Persistently Neutropenic Rabbits. Antimicrob. Agents Chemother..

[66] Fernández-Torres B., Inza I., Guarro J. (2003). In Vitro Activities of the New Antifungal Drug Eberconazole and Three Other Topical Agents against 200 Strains of Dermatophytes. J. Clin. Microbiol..

[67] Forouzesh A., Foroushani S.S., Forouzesh F., Zand E. (2019). Reliable Target Prediction of Bioactive Molecules Based on Chemical Similarity without Employing Statistical Methods. Front. Pharmacol..

[68] Seidel J.S., Harmatz P., Visvesvara G.S., Cohen A., Edwards J., Turner J. (1982). Successful treatment of primary amebic meningoencephalitis. N. Engl. J. Med..

[69] Debnath A., Calvet C.M., Jennings G., Zhou W., Aksenov A., Luth M.R., Abagyan R., Nes W.D., McKerrow J.H., Podust L.M. (2017). CYP51 Is an Essential Drug Target for the Treatment of Primary Amoebic Meningoencephalitis (PAM). PLoS Negl. Trop. Dis..

[70] Nayak S.U., Griffiss J.M., Blumer J., O’Riordan M.A., Gray W., McKenzie R., Jurao R.A., An A.T., Le M., Bell S.J. (2017). Safety, Tolerability, Systemic Exposure, and Metabolism of CRS3123, a Methionyl-tRNA Synthetase Inhibitor Developed for Treatment of Clostridium difficile, in a Phase 1 Study. Antimicrob. Agents Chemother..

[71] Ricci F., Tedeschi A., Morra E., Montillo M. (2009). Fludarabine in the Treatment of Chronic Lymphocytic Leukemia: A Review. Ther. Clin. Risk Manag..

[72] Amsden G.W. (1996). Erythromycin, Clarithromycin, and Azithromycin: Are the Differences Real?. Clin. Ther..

[73] Al Hossain A.S.M.M., Sil B.C., Iliopoulos F., Lever R., Hadgraft J., Lane M.E. (2019). Preparation, Characterisation, and Topical Delivery of Terbinafine. Pharmaceutics.

[74] Yamaguchi H. (2016). Potential of Ravuconazole and Its Prodrugs as the New Oral Therapeutics for Onychomycosis. Med. Mycol. J..

[75] Zhou W., Debnath A., Jennings G., Hahn H.J., Vanderloop B.H., Chaudhuri M., Nes W.D., Podust L.M. (2018). Enzymatic Chokepoints and Synergistic Drug Targets in the Sterol Biosynthesis Pathway of Naegleria Fowleri. PLoS Pathog..

[76] Ryder N.S. (1992). Terbinafine: Mode of Action and Properties of the Squalene Epoxidase Inhibition. Br. J. Dermatol..

[77] Nulens E., Eggink C., Rijs A.J.M.M., Wesseling P., Verweij P.E. (2003). Keratitis Caused by Scedosporium Apiospermum Successfully Treated with a Cornea Transplant and Voriconazole. J. Clin. Microbiol..

[78] Schmitt-Hoffmann A.H., Kato K., Townsend R., Potchoiba M.J., Hope W.W., Andes D., Spickermann J., Schneidkraut M.J. (2017). Tissue Distribution and Elimination of Isavuconazole Following Single and Repeat Oral-Dose Administration of Isavuconazonium Sulfate to Rats. Antimicrob. Agents Chemother..

[79] Lamoth F., Mercier T., André P., Pagani J.L., Pantet O., Maduri R., Guery B., Decosterd L.A. (2019). Isavuconazole Brain Penetration in Cerebral Aspergillosis. J. Antimicrob. Chemother..

[80] Sharma M., Sudhan S.S., Sharma S., Megha K., Nada R., Khurana S. (2017). Osteo-Cutaneous Acanthamoebiasis in a Non-Immunocompromised Patient with a Favorable Outcome. Parasitol. Int..

[81] Van Tyle J.H. (1984). Ketoconazole. Mechanism of action, spectrum of activity, pharmacokinetics, drug interactions, adverse reactions and therapeutic use. Pharmacotherapy.

[82] Iwatani W., Arika T., Yamaguchi H. (1993). Two Mechanisms of Butenafine Action in Candida Albicans. Antimicrob. Agents Chemother..

[83] Torres-Rodríguez J.M., Mendez R., López-Jodra O., Morera Y., Espasa M., Jimenez T., Lagunas C. (1999). In Vitro Susceptibilities of Clinical Yeast Isolates to the New Antifungal Eberconazole Compared with Their Susceptibilities to Clotrimazole and Ketoconazole. Antimicrob. Agents Chemother..

[84] Mercer E.I. (1991). Morpholine Antifungals and Their Mode of Action. Biochem. Soc. Trans..

[85] Polak A. (1988). Mode of Action of Morpholine Derivatives. Ann. N. Y. Acad. Sci..

[86] Mcneely W., Spencer C.M. (1998). Butenafine. Drugs.

[87] Bezerra-Souza A., Fernandez-Garcia R., Rodrigues G.F., Bolas-Fernandez F., Laurenti M.D., Passero L.F., Lalatsa A., Serrano D.R. (2019). Repurposing Butenafine as an Oral Nanomedicine for Visceral Leishmaniasis. Pharmaceutics.

[88] Xu Y., Pang G.R., Zhao D.Q., Gao C.W., Zhou L.T., Sun S.T., Wang B.L., Chen Z.J. (2010). Activity of butenafine against ocular pathogenic filamentous fungi in vitro. Zhonghua Yan Ke Za Zhi.

[89] Lopez S.E., Salazar J. (2013). Trifluoroacetic acid: Uses and recent applications in organic synthesis. J. Fluor. Chem..

[90] Mcdonnell G., Russell A.D. (1999). Antiseptics and Disinfectants: Activity, Action, and Resistance. Clin. Microbiol. Rev..

[91] Allas Ü., Toom L., Selyutina A., Mäeorg U., Medina R., Merits A., Rinken A., Hauryliuk V., Kaldalu N., Tenson T. (2016). Antibacterial Activity of the Nitrovinylfuran G1 (Furvina) and Its Conversion Products. Sci. Rep..

[92] Siddiqui R., Aqeel Y., Khan N.A. (2016). The Development of Drugs against Acanthamoeba Infections. Antimicrob. Agents Chemother..

[93] Alizadeh H., Neelam S., Cavanagh H.D. (2009). Amoebicidal Activities of Alexidine Against 3 Pathogenic Strains of Acanthamoeba. Eye Contact Lens..

[94] Heaselgrave W., Hamad A., Coles S., Hau S. (2019). In Vitro Evaluation of the Inhibitory Effect of Topical Ophthalmic Agents on Acanthamoeba Viability. Transl. Vis. Sci. Technol..

[95] Rice C.A., Campbell S.J., Bisson C., Owen H.J., Sedelnikova S.E., Baker P.J., Rice D.W., Henriquez F.L., Roberts C.W. (2018). Structural and Functional Studies of Histidine Biosynthesis in Acanthamoeba Spp. Demonstrates a Novel Molecular Arrangement and Target for Antimicrobials. PLoS ONE.

[96] Zhang J.H., Chung T.D., Oldenburg K.R. (1999). A Simple Statistical Parameter for Use in Evaluation and Validation of High Throughput Screening Assays. J. Biomol. Screen..

