# Supplementary material for: Discovery of Anti-Amoebic Inhibitors from Screening the MMV Pandemic Response Box on Balamuthia mandrillaris, Naegleria fowleri, and Acanthamoeba castellanii
Source: Pathogens. 2020 Jun 16;9(6):476. doi: 10.3390/pathogens9060476 (PMC7344389; doi:10.3390/pathogens9060476)
Supplement: Supplementary file 1 [file pathogens-09-00476-s001.pdf]

pathogens

MDPI

Article

Discovery of anti-amoebic inhibitors from screening the MMV Pandemic Response Box on *Balamuthia mandrillaris, Naegleria fowleri* and *Acanthamoeba castellanii*

Christopher A. Rice[1,2,Δ,†,*], Emma V. Troth[2,3,†], A. Cassiopeia Russell[2,3,†], and Dennis E. Kyle[1,2,3,*]

1 Department of Cellular Biology, University of Georgia, Athens, Georgia, USA.
2 Center for Tropical and Emerging Global Diseases, Athens, Georgia, USA.
3 Department of Infectious Diseases, University of Georgia, Athens, Georgia, USA.
Δ Current address: Department of Pharmaceutical and Biomedical Sciences, College of Pharmacy, University of Georgia, Athens, Georgia, USA.
† These authors contributed equally to this work.
* Correspondence: dennis.kyle@uga.edu (D.E.K) and christopher.rice@uga.edu (C.A.R)

Received: 12 May 2020; Accepted: date; Published: date

Supplemental Figure 1. Morphology and density of *Balamuthia mandrillaris* trophozoites in a negative control well after 72 hr (16,000 amoebae/well).

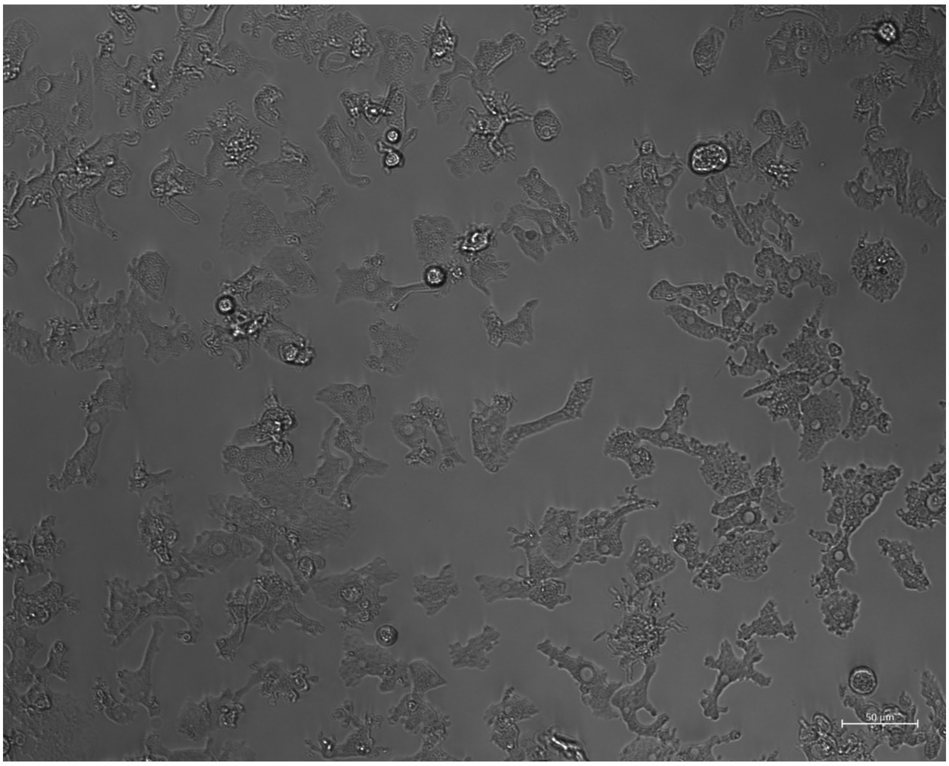

© 2020 by the authors. Submitted for possible open access publication under the terms and conditions of the Creative Commons Attribution (CC BY) license (http://creativecommons.org/licenses/by/4.0/).
